# Supplementary material for: Titanium Dioxide Nanoparticles Modulate Systemic Immune Response and Increase Levels of Reduced Glutathione in Mice after Seven-Week Inhalation
Source: Nanomaterials (Basel). 2023 Feb 18;13(4):767. doi: 10.3390/nano13040767 (PMC9964099; doi:10.3390/nano13040767)
Supplement: Supplementary file 1 [file nanomaterials-13-00767-s001.zip › nanomaterials-2071791-supplementary.pdf]

nanomaterials

Supplementary Materials

Titanium dioxide nanoparticles suppress the systemic immune response in mice after seven-week inhalation

Miroslava Lehotska Mikusova [1*†], Milena Busova [2*†], Jana Tulinska [1], Vlasta Masanova [1], Aurelia Liskova [1], Iveta Uhnakova [1], Maria Dusinska [3], Zora Krivosikova [1], Eva Rollerova [4], Radka Alacova [4], Ladislava Wsolova [4], Mira Horvathova [1], Michaela Szabova [1], Norbert Lukan [1], Zbynek Vecera [5], Pavel Couflik [5], Kamil Krumal [5], Lukas Alexa [5], Vojtech Thon [6], Pavel Piler [6], Marcela Buchtova [7], Lucie Vrlikova [7], Pavel Moravec [8], Dusan Galanda [9] and Pavel Mikuska [5*]

[1] Faculty of Medicine, Slovak Medical University, 833 03 Bratislava, Slovakia
[2] Institute of Hygiene and Epidemiology, First Faculty of Medicine, Charles University and General University Hospital in Prague, 121 08 Prague, Czech Republic
[3] Health Effects Laboratory, Norwegian Institute for Air Research, 2027 Kjeller, Norway
[4] Faculty of Public Health, Slovak Medical University, 833 03 Bratislava, Slovakia
[5] Department of Environmental Analytical Chemistry, Institute of Analytical Chemistry of the Czech Academy of Sciences, 602 00 Brno, Czech Republic
[6] RECETOX, Faculty of Science, Masaryk University, 625 00 Brno, Czech Republic
[7] Laboratory of Molecular Morphogenesis, Institute of Animal Physiology and Genetics, Czech Academy of Sciences, 602 00 Brno, Czech Republic
[8] Aerosol Chemistry and Physics Research Group, Institute of Chemical Process Fundamentals of the Czech Academy of Sciences, 165 00 Prague, Czech Republic
[9] Department of Nuclear Chemistry, Faculty of Natural Sciences, Comenius University in Bratislava, 826 45 Bratislava, Slovakia
* Correspondence: miroslava.mikusova@szu.sk (M.L.M.); milena.busova@lf1.cuni.cz (Mi.B.); mikuska@iach.cz (P.Mi.)
† First author

Table S1. In vivo inhalation studies investigated the adverse effects of TiO_2 NPs on systemic immune response and oxidative stress

Model / Animal	Administration mode	Particulate size	Dose and duration of treatment	Outcomes	Authors	Ref. No.
mice	whole-body inhalation chambers	29.6 nm	**0.00167 and 0.1308 mg TiO_2/m^3,** continuously for 7 weeks **i.e. 0.012 and 0.958 mg/kg b.w.**	- decreased percentage of T lymphocytes in spleen in both dose groups<br>- decreased percentages of T-helper lymphocytes in spleen at the low dose<br>- stimulated phagocytic activity of monocytes in blood at the low dose<br>- decreased levels of IL-4 and IL-18 in spleen at the high dose<br>- increased levels of GSH and GSH/GSSG ratio	Our study	
mice	whole-body inhalation chambers	20 nm (anatase)	**6.34 ± 0.22 mg TiO_2/m^3,** 8 hours/day, for 3 weeks	- decreased white blood cell count<br>- increased platelet and reticulocyte counts	Yin et al. 2014	[7]
mice	whole-body inhalation exposure system	30 nm (anatase)	**2, 8 or 32 mg/m^3 of TiO_2 NPs,** 6 hours/day, 5 days/week for 26 weeks	- decreased red blood cell count and hematocrit value in males<br>- increased MCV and MCH<br>- decreased white blood cell count in females	Yamano et al. 2021	[53]
mice	intratracheal instillation	20 nm	**5, 20, and 50 mg/kg b.w.,** single dose, animals sacrificed at 1, 7, 14 days after treatment	- induced pro-inflammatory cytokines, Th1-type cytokines and Th2-type cytokines followed by B cell proliferation,<br>- dose-dependently elevated levels of the pro-inflammatory cytokines IL-1, TNF- α, and IL-6<br>- dose-dependently elevated levels of the Th1 cytokines IL-12 and IFN- γ, and the Th2 cytokines IL4, IL-5 and IL-10<br>- increased numbers of B lymphocytes in both spleen and blood	Park et al. 2009	[35]
mice	intratracheal instillation	10.5 x 50-60 nm (nanorods) (rutile)	**0.162 mg TiO_2/kg b.w.,** single dose	- no effect on hematological parameters	Modrzynska et al. 2021	[76]
mice	intranasal instillation	80 nm (rutile)	**500 µg TiO_2 NP suspension,** every other day for 30 days	- no effect on GSH content, GPx, GST, and SOD activities in brain<br>- increased catalase activity in brain	Wang et al. 2008	[73]

mice	intranasal instillation	80 nm (rutile)	**500 µg TiO_2 NP suspension,** applied every other day, for 2, 10, 20 and 30 days	- increased activities of GPx, GST and SOD and GSH level in brain at 10 days postexposure<br>- no effect on activities of GPx, GST and SOD and GSH level in brain at 20 and 30 days postexposure<br>- increased MDA in brain at 30 days postexposure	Wang et al. 2008	[74]
rats	intratracheal instillation	4-6 nm nanorods (primary diameter) (rutile)	**1 and 5 mg/kg b.w.,** single dose	- increased number of monocytes and granulocytes in the blood in a dose-dependent manner<br>- decreased number of platelets<br>- no effect on the number of lymphocytes	Nemmar et al. 2008	[33]
rats	intratracheal instillation	21 nm (80% anatase, 20% rutile)	**0.5, 4, and 32 mg/kg b.w.,** twice a week for 4 weeks	- increased proliferation of T cells and B cells following mitogen stimulation<br>- enhanced natural killer (NK) cell killing activity in spleen<br>- increased number of B cells in blood<br>- no significant changes of Th1-type cytokines (IL-2 and INF-γ) and Th2-type cytokines (TNF-α and IL-6)	Fu et al. 2014	[8]
rats	intratracheal instillation	23 nm (anatase, rutile)	**3.5** and **17.5 mg/kg b.w.** once every 2 days for 5 weeks	**Blood:**<br>- increased MDA, NO, NEUT, and IL-1<br>- decreased GSH, WBC, IFN-γ, and TNF-α<br>**Spleen:**<br>- increased MDA<br>**Liver:**<br>- decreased GSH level and SOD activity<br>- increased MDA<br>**Thymus:**<br>- dose-dependently decreased SOD activity	Liu et al. 2015	[62]
rats	intratracheal instillation	23 nm (anatase, rutile)	**0.5, 2.5 and 10 mg/kg b.w.,** subdivided into 3 instillations at 4-day intervals, animals sacrificed 2 hours and 35 days after 3 treatments	- no effect on total glutathione content in plasma<br>- oxidized glutathione levels in blood were below the detection range	Relier et al. 2017	[72]
rats	intranasal instillation	7 x 80 nm Fe–TiO_2 nanorods (rutile)	**1 and 5 mg/kg b.w.,** single dose, animals sacrificed 24 hours treatment	- increased plasma levels IL-6<br>- dose-dependently decreased plasma SOD and GSH activities<br>- decreased number of platelets	Nemmar et al. 2011	[77]

rats	intranasal instillation	21 nm (75% anatase, 25% rutile)	**5 mg TiO_2/kg b.w.,** single dose, animals sacrificed at 1, 2, 8, 16, 30, and 90 days after treatment	- NP-induced immunoactivating and proinflammatory activity in blood: ***Days 1–2 post-exposure,*** - elevated levels of IL-2, IL-4, IL-6, IL-10, and IFN-γ ***Days 2–8 post-exposure,*** - increased CINC-1 levels ***Day 16 post-exposure,*** - increased TNF-α	Gustafsson et al. 2011	[34]
rats	nose-only inhalation	21 nm (80% anatase, 20% rutile)	**10 mg/m^3 of TiO_2 NPs,** for 21 days, 6 h/day, animals sacrificed at 3, 28, and 90 days after treatment	**Day 3 post-exposure:** - no significant changes in hematology. **Day 28 post-exposure:** - reduced white blood cell and lymphocyte counts **Day 90 post-exposure:** - reduced white blood cell and lymphocyte counts - reduced number of segmented neutrophils	Eydner et al. 2012	[78]

Abbreviations

GSH: reduced form of glutathione

GSSG: oxidized form of glutathione

GST: glutathione S-transferase

GPx: glutathione peroxidase

IFN-γ: interferon-γ

IL: interleukin

MCH: mean corpuscular haemoglobin

MCV: mean corpuscular volume (as red blood cell indices)

MDA: malondialdehyde

NEUT: neutrophils

SOD: superoxide dismutase

TiO_2 NPs: titanium dioxide nanoparticles

TNF-α: tumor necrosis factor α

WBC: white blood cells

References

7. Yin, J.; Kang, C.; Li, Y.; Li, Q.; Zhang, X.; Li, W. Aerosol inhalation exposure study of respiratory toxicity induced by 20 nm anatase titanium dioxide nanoparticles. *Toxicol. Res.* **2014**, *3*, 367–374. doi:10.1039/C4TX00040d.

8. Fu, Y.; Zhang, Y.; Chang, X.; Zhang, Y.; Ma, S.; Sui, J.; Yin, L.; Pu, Y.; Liang, G. Systemic immune effects of titanium dioxide nanoparticles after repeated intratracheal instillation in rat. *Int. J. Mol. Sci.* **2014**, *15*, 6961–6973. doi:10.3390/ijms15046961.

33. Nemmar, A.; Melghit, K.; Ali, B.H. The acute proinflammatory and prothrombotic effects of pulmonary exposure to rutile TiO_2 nanorods in rats. *Exp. Biol. Med. (Maywood)* **2008**, *233*, 610–619. doi:10.3181/0706-RM-165.

34. Gustafsson, Å.; Lindstedt, E.; Elfsmark, L.S.; Bucht, A. Lung exposure of titanium dioxide nanoparticles induces innate immune activation and long-lasting lymphocyte response in the Dark Agouti rat. *J. Immunotoxicol.* **2011**, *8*, 111–121. doi:10.3109/1547691X.2010.546382.

35. Park, E.J.; Yoon, J.; Choi, K.; Yi, J.; Park, K. Induction of chronic inflammation in mice treated with titanium dioxide nanoparticles by intratracheal instillation. *Toxicology* **2009**, *260*, 37–46. doi:10.1016/j.tox.2009.03.005.

53. Yamano, S., Takeda, T., Goto, Y., Hirai, S., Furukawa, Y., Kikuchi, Y., Kasai, T., Misumi, K., Suzuki, M., Takanobu K., et al. Lack of pulmonary fibrogenicity and carcinogenicity of titanium dioxide nanoparticles in 26-week inhalation study in rasH2 mouse model. bioRxiv **2021**, 2021.12.23.473959; doi:10.1101/2021.12.23.473959.

62. Liu, H.L.; Yang, H.L.; Lin, B.C.; et al. Toxic effect comparison of three typical sterilization nanoparticles on oxidative stress and immune inflammation response in rats. *Toxicol. Res. (Camb)* **2015**, *4*, 486–493. doi:10.1039/C4TX00154K.

72. Relier, C.; Dubreuil, M.; Lozano Garcìa, O.; Cordelli, E.; Mejia, J.; Eleuteri, P.; Robidel, F.; Loret, T.; Pacchierotti, F.; Lucas, S.; Lacroix, G.; Trouiller, B. Study of TiO_2 P25 Nanoparticles Genotoxicity on Lung, Blood, and Liver Cells in Lung Overload and Non-Overload Conditions After Repeated Respiratory Exposure in Rats. *Toxicol. Sci.* **2017**, *156*, 527–537. doi:10.1093/toxsci/kfx006.

73. Wang, J.; Chen, C.; Liu, Y.; Jiao, F.; Li, W.; Lao, F.; Li, Y.; Li, B.; Ge, C.; Zhou, G.; et al. Potential neurological lesion after nasal instillation of TiO_2 nanoparticles in the anatase and rutile crystal phases. *Toxicol. Lett.* **2008**, *183*, 72–80. https://doi.org/10.1016/j.toxlet.2008.10.001.

74. Wang, J.; Liu, Y.; Jiao, F.; Lao, F.; Li, W.; Gu, Y.; Li, Y.; Ge, C.; Zhou, G.; Li, B.; Zhao, Y.; Chai, Z.; Chen, C. Time-dependent translocation and potential impairment on central nervous system by intranasally instilled TiO_2 nanoparticles. *Toxicology* **2008**, *254*, 82–90. doi:10.1016/j.tox.2008.09.014.

76. Modrzynska, J.; Mortensen, A.; Berthing, T.; Ravn-Haren, G.; Szarek, J.; Saber, A.T.; Vogel, U. Effect on Mouse Liver Morphology of CeO_2, TiO_2 and Carbon Black Nanoparticles Translocated from Lungs or Deposited Intravenously. *Appl. Nano* **2021**, *2*, 222–241. doi:10.3390/applnano2030016.

77. Nemmar, A.; Melghit, K.; Al-Salam, S.; Zia, S.; Dhanasekaran, S.; Attoub, S.; Al-Amri, I.; Ali, B.H. Acute respiratory and systemic toxicity of pulmonary exposure to rutile Fe-doped TiO(2) nanorods. *Toxicology* **2011**, *279*, 167–175. doi:10.1016/j.tox.2010.10.007.

78. Eydner, M.; Schaudien, D.; Creutzenberg, O.; Ernst, H.; Hansen, T.; Baumgärtner, W.; Rittinghausen, S. Impacts after inhalation of nano- and fine-sized titanium dioxide particles: morphological changes, translocation within the rat lung, and evaluation of particle deposition using the relative deposition index. *Inhal. Toxicol*. **2012**, *24*, 557–569. doi:10.3109/08958378.2012.697494.
